# Effects of exercise-based home pulmonary rehabilitation on patients with chronic obstructive pulmonary disease: An overview of systematic review

**DOI:** 10.1371/journal.pone.0277632

**Published:** 2022-11-17

**Authors:** Jiang Zheng, Zhi Zhang, Ruijuan Han, Hongxia Zhang, Jie Deng, Meimei Chai

**Affiliations:** 1 Department of Respiratory and Critical Care Medicine, The 940th Hospital of Joint Logistics Support Force of Chinese People’s Liberation Army, Lanzhou, China; 2 Anesthesia and Operating Department, Gansu Provincial Maternity and Child-Care Hospital, Lanzhou, China; 3 School of Nursing, Gansu University of Chinese Medicine, Lanzhou, China; 4 School of Nursing, Lanzhou University, Lanzhou, China; Mugla Sitki Kocman Universitesi, TURKEY

## Abstract

**Background:**

Clinical research on exercise-based home pulmonary rehabilitation (HPR) effectiveness in chronic obstructive pulmonary disease (COPD) treatment is rising, as are associated systematic reviews/meta-analyses (SRs/MAs). However, different SRs/MAs vary in outcome indicators, analysis methodologies, literature quality, and findings. This overview aimed to describe the findings of these SRs/MAs and assess their methodological quality.

**Methods:**

From inception until April 2022, we searched PubMed, Web of Science, Cochrane Library, EMBASE, China National Knowledge Infrastructure (CNKI), and Wan Fang. Two researchers searched these SRs/MAs separately, collected the data, and cross-checked it using predetermined rules. The Assessing the Methodological Quality of Systematic Reviews 2 (AMSTAR 2) was used to evaluate the methodological quality of each contained SR/MA. The evidence was assessed using the Preferred Reporting Items for Systematic Reviews and Meta-Analyses 2009 (PRISMA-2009). The Grades of Recommendation, Assessment, Development, and Evaluation (GRADE) was used to determine the validity of the results.

**Results:**

A total of 433 records were found, with 44 chosen for full-text review. There were 11 SRs/MAs that matched the inclusion criteria. Our overview included studies published from 2010 to 2022. According to the AMSTAR 2 tool, one had low methodological quality, while the other 10 SRs/MAs had very low quality. The PRISMA statement revealed a low rate of complete reporting for eight items. The GRADE tool, on the other hand, revealed that the evidence quality for most outcomes was very low to moderate.

**Conclusion:**

According to current research, exercise-based HPR may benefit COPD patients. Nevertheless, this finding is restricted by the low quality of the included SRs/MAs. And more high-quality and large-sample studies are needed in the future.

**Prospero:**

ID: CRD42022322768. https://www.crd.york.ac.uk/prospero/#recordDetails

## 1. Introduction

Chronic obstructive pulmonary disease is a complicated respiratory disease defined by recurring, persistent, and irreversible obstructive airflow restriction [1]. Patients with COPD may have less physical activity, resulting in muscular deconditioning, increased difficulty with dyspnea, and a lower quality of life [2]. According to the World Health Organization, COPD is now the third leading cause of death worldwide [3]. As we face an aging society and an increase in various risk factors for COPD, COPD loads are predicted to rise in the future decades [4]. COPD has become a significant public health issue.

Pulmonary rehabilitation (PR) has become the gold standard in COPD treatment. Pulmonary rehabilitation is a comprehensive intervention based on a thorough assessment of the condition of patients with respiratory diseases, which mainly includes exercise, respiratory training, education of patients, and intervention of poor lifestyle habits [5]. PR aims to improve the physical and psychological status of patients with respiratory diseases and long-term compliance with health-promoting behaviors. PR specifics include but are not limited to sports, self-management, dietary guidance, smoking cessation, education, and behavior adjustment [6]. PR has substantial evidence to demonstrate improved anxiety and depression and fewer hospitalizations and hospital days in COPD patients [7].

The exercise-based pulmonary rehabilitation program is an essential part of COPD care. There is solid evidence that it can increase exercise capacity, reduce symptoms, including dyspnea and fatigue, and enhance health-related quality of life (HRQoL) [8, 9]. According to the British Thoracic Society guideline on pulmonary rehabilitation in adults, a rehabilitation program should include supervised, individualized, progressive exercise training [6]. COPD patients might benefit from various exercises [10–12].

Traditional PR is performed by patients in an outpatient setting in a hospital or other medical facility [13]. However, absenteeism and loss of follow-up in pulmonary rehabilitation programs are widespread due to a lack of planning, travel and transportation challenges, and other health concerns [14]. The current COVID-19 pandemic has increased the number of patients with PR indications, and it has also raised the strain on PR services by increasing treatment obstacles due to cross-infection concerns [15].

HPR is performed in a nonmedical context, such as the patient’s home or community, and requires less space, time, and rehabilitation equipment [16]. HPR is more convenient, less expensive, and more suitable for individuals with significant motor disabilities and mobility limits than hospital-based treatments [17]. It could be a viable alternative to traditional inpatient and outpatient pulmonary rehabilitation [18]. Numerous SRs/MAs on the benefits of training-based home pulmonary rehabilitation in COPD patients have been published in recent years. However, a large number of published SRs/MAs are of variable methodological quality, with varying findings and limitations. Therefore, it is necessary to summarize these SRs/MAs.

A systematic review overview aims to compare, summarize, and synthesize results from several SRs/MAs [19]. Overviews benefit decision-makers by synthesizing the findings of the included studies and providing them with readily available evidence [20]. Therefore, the goal of this overview was to synthesize information from SRs/MAs to summarize the implications of home-based PR for COPD patients.

## 2. Methods

We followed the PRISMA statement [21], the Cochrane Handbook [22], and the PRIOR statement [23]. This overview was registered with PROSPERO (no. CRD42022322768).

### 2.1. Search strategy

The included studies were available in Chinese or English only. We systematically searched PubMed, Web of Science, Cochrane Library, Embase, CNKI, and Wan Fang databases from inception to April 2022. The following keywords were used: exercise, pulmonary rehabilitation, home, COPD, and meta-analysis. In addition, we carefully checked the references of all included research to confirm that the search was comprehensive. S1 Table contains the detailed search tactics and steps.

### 2.2. Inclusion and exclusion criteria

#### 2.2.1. Inclusion criteria

1. Types of Included Reviews
  SRs/MAs are based on randomized controlled trials (RCTs) or non-randomized controlled trials (non-RCTs), with or without meta-analysis.
2. Participants
  Adults diagnosed with chronic obstructive pulmonary disease (mild to very severe) were selected. The diagnostic criteria are based on the Global Initiative for Chronic Obstructive Lung Disease (GOLD) guidelines [1], the European Respiratory Society [24], the American Thoracic Society [25], and the British Thoracic Society [26].
3. Interventions
  The interventions refer to exercise-based home pulmonary rehabilitation, with/without usual care, such as walking, running, aerobic training, endurance training, resistance training, interval training, upper or lower limb training, etc.
4. Comparators
  The comparator was usual care, no treatment, or in/outpatient exercise-based pulmonary rehabilitation.
5. Outcomes
  We included HRQoL, exercise capacity, dyspnea, and pulmonary function as outcome indicators. HRQoL was measured by St George’s Respiratory Questionnaire (SGRQ) or Chronic Respiratory Disease Questionnaire (CRQ). The findings of a 6-minute walk distance/test (6MWD/6MWT), an incremental shuttle-walk test (ISWT), a shuttle-walk test (SWT), or an endurance shuttle-walk test (EWST) were used to determine exercise capacity. Dyspnea was measured by the Borg scale, Medical Research Council (MRC), modified British Medical Research Council (mMRC), or CRQ-dyspnea. Pulmonary function indicators included FEV1, FVC, and FEV1/FVC.

#### 2.2.2. Exclusion criteria

Duplicate publications, plans, reviews, conference abstracts, editorials, and studies for which the full text was unavailable were excluded.

### 2.3. Literature selection

Two authors used a predefined standardized search technique to search the database. All search results were integrated into Endnote X9 software to eliminate duplicate content. Two authors examined the title and abstract independently and excluded literature that did not fit the literature’s inclusion and exclusion criteria. The full text was then read again to identify the final literature that would be included. A third author served as a judge to resolve any differences.

### 2.4. Data extraction

Based on a predesigned Excel spreadsheet, two authors extracted information separately. The information extraction included first author, publication year, type and the number of included studies, participants, interventions, comparisons, outcomes, tools for methodological quality assessment, and critical findings. A third author acted as a referee to resolve any disagreements.

### 2.5. Review quality assessment

Two authors evaluated the included SRs/MAs in the study separately. After concluding the evaluation, two authors double-checked the results. A third author served as a judge to resolve any differences.

The methodological quality of the included SRs/MAs was assessed using the AMSTAR-2 instrument [27]. There are 16 items in total, seven of which are crucial. Each item was given a “yes,” “partial yes,” “no,” or “not conducted”. Ultimately, the quality of each SR/MA was graded (four levels of high, medium, low, or very low) according to seven critical items.

The quality of each SR/MA report was assessed using the PRISMA checklist [28]. It consists of 27 elements that focus on each SR/MA’s reported methods and outcomes. “Yes,” “partial yes,” or “no” were used to respond to each item. Each item’s ultimate completion was reported as a ratio.

The GRADE [29] was used to assess the quality of the primary outcomes of the SRs/MAs included in the overview. Five key elements (limitations, inconsistency, indirectness, imprecision, and publication bias) were used to divide the quality of evidence into four grades (high, moderate, low, and very low).

### 2.6. Overlap calculation

The degree of duplication of the original literature for SRs/MAs was assessed by creating citation matrices for SRs/MAs and calculating the “corrected covered area” (CCA) [30]. The formula was calculated as CCA = (n-r)/(rc-r), where “n” is all original studies included in SRs/MAs, “r” is all original studies included in SRs/MAs after de-duplication, and “c” is the number of studies included in the overview this time. The calculation result “0–5” indicates slight overlap, “6–10” indicates moderate overlap, “11–15” indicates high overlap, and “>15” indicates very high overlap.

## 3. Results

### 3.1. Search results

**Fig 1:**
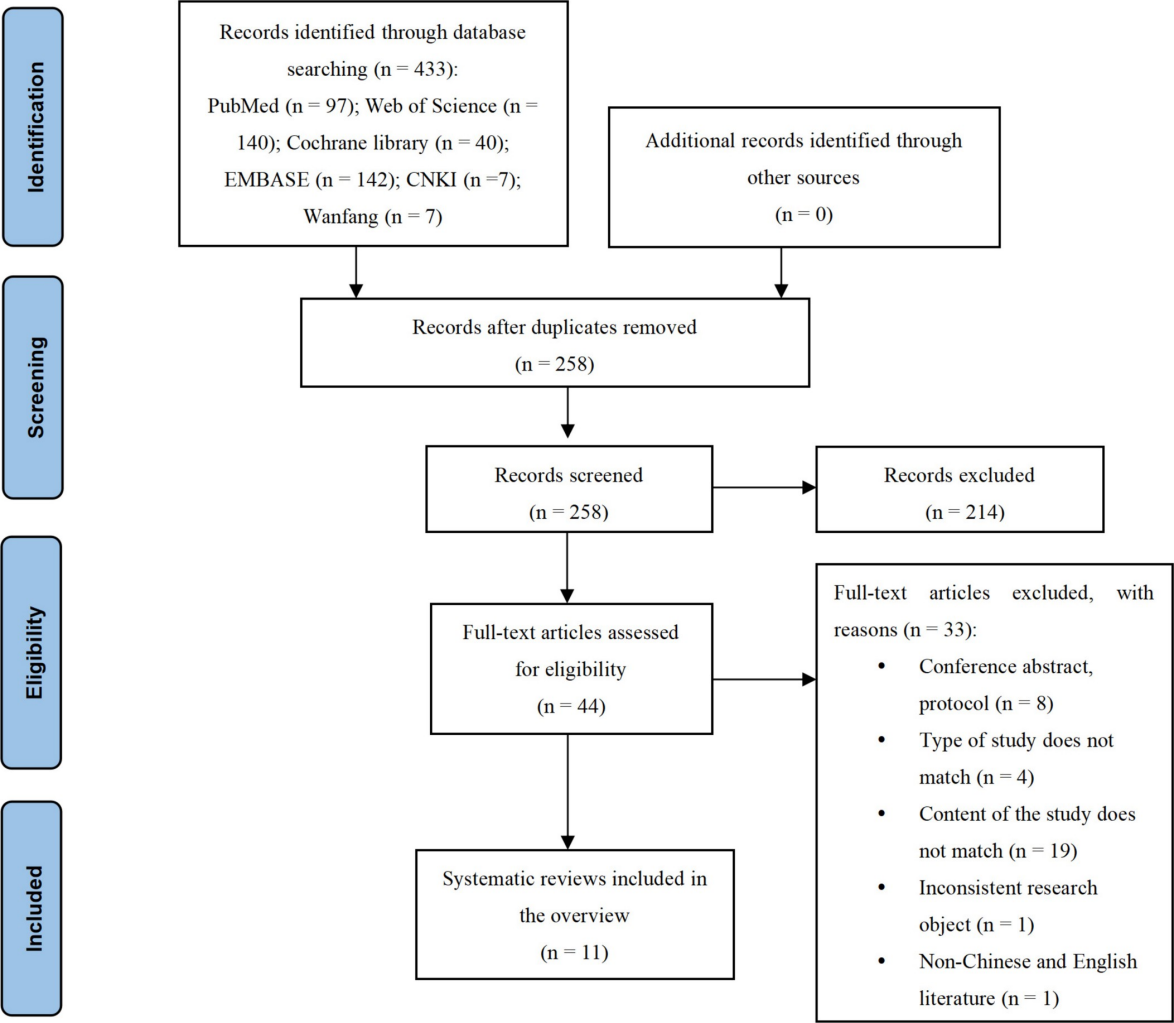
Flow diagram of study selection.

433 published studies were discovered through the database search, with 175 repeated studies removed. A total of 214 publications were eliminated after reviewing the titles and abstracts. The full papers of 44 articles were downloaded when they were deemed eligible. Thirty-three papers were excluded after a full-text examination. Ultimately, this overview contained 11 SRs/MAs. Fig 1 shows the study screening process. S2 Table shows a list of excluded publications and the grounds for their exclusion.

### 3.2. Features of included studies

The detailed features of each of those SRs/MAs are summarized in Table 1. The SRs/MAs in this overview were published between 2010 and 2022. There were 11 SRs/MAs in all, seven in English and four in Chinese. The total number of participants in SRs/MAs ranged from 464 to 2352, and the number of RCTs ranged from 9 to 23. For methodological quality assessment, seven articles used the Cochrane Risk of Bias instrument, one used the Jadad scale, one used the PEDro scale, one used the Quality Assessment Tool for Quantitative Studies, and the other article did not mention any particular tool. The intervention group used exercise-based home pulmonary rehabilitation, including endurance exercise training, aerobic training, strength training, breathing gymnastics, etc. HPR based on activity was typically four weeks or longer, two sessions per week or more, and at least 30 minutes per session. The control group received usual care, conventional medication, no intervention, or outpatient/inpatient PR. Of the 11 included studies, six compared exercise-based HPR with usual care, standard medical care, or no intervention [32–35, 38, 40], three compared exercise-based HPR with outpatient or center-based PR [36, 37, 39] and two included both comparisons [16, 31].

**Table 1:** Characteristics of the included reviews.

First author and year	Type and number of included studies	Total number of participants	Intervention	Comparisons	Outcomes	Quality assessment tool	Authors conclusions
Vieira (2010) [31]	N = 12 12 RCTs	728	Exercise-based HPR: LLE, walking, cycling, stair climbing, endurance training, down a stair and sitting to standing from a chair	Hospital-based PR, Standard medical care, No intervention	1) HRQoL: CRQ, SGRQ 2) Exercise capacity: 6MWD, SWT 3) Dyspnea: MRC, Borg	PEDro	Home-based pulmonary rehabilitation can potentially improve HRQoL and exercise capacity compared to standard care.
Wang (2013) [32]	N = 15 15 RCTs	518	Exercise-based HPR: LLE, ULE	Usual care	1) HRQoL: CRQ 2) Exercise capacity: 6MWD 3) Dyspnea: CRQ-D	Not report	Home pulmonary rehabilitation can improve exercise tolerance, dyspnea, and fatigue in patients with COPD.
Liu (2014) [33]	N = 18 18 RCTs	733	Exercise-based HPR: LLE, ULE, walking, cycling, resistance exercise, endurance training	Conventional community care without rehabilitation	1) HRQoL: CRQ, SGRQ 2) Exercise capacity: 6MWD 3) Dyspnea: Borg, CRQ-D 4) Pulmonary function: FEV_1_/FVC	Cochrane criteria	Home-based pulmonary rehabilitation programs represent effective therapeutic intervention approaches for relieving dyspnea status and improving exercise capacity, pulmonary functions, and HRQoL among COPD populations.
Liu (2016) [34]	N = 13 13 RCTs	464	Exercise-based HPR: LLE, ULE	Conventional community care	1) HRQoL: CRQ, SGRQ 2) Exercise capacity: 6MWD 3) Dyspnea: Borg, CRQ-D	Cochrane criteria	Home pulmonary rehabilitation can effectively improve health-related quality of life and physical function in COPD patients.
Neves (2016) [16]	N = 23 23 RCTs	1258	Exercise-based HPR: LLE, ULE, walking, stretching Exercise-based community PR: LLE, ULE, rowing machine, climbing stairs, swimming, skating, and bicycling	Standard medical care, Usual care, OPR	1) HRQoL: CRQ, SGRQ 2) Exercise capacity: 6MWT, ISWT 3) Dyspnea: MRC, CRQ-D	Cochrane criteria	Compared to a control group, home or community-based PR improved functional capacity, decreased dyspnea sensation, and improved quality of life.
Li (2017) [35]	N = 14 14 RCTs	495	Exercise-based HPR: LLE, ULE	Usual care	1) HRQoL: CRQ, SGRQ 2) Exercise capacity: 6MWD, SWT 3) Dyspnea: Borg, MRC, CRQ-D 4) Pulmonary function: FEV_1_/FVC	Cochrane criteria	Home pulmonary rehabilitation can effectively improve quality of life, exercise capacity, and dyspnea symptoms in stable COPD patients but has no significant improvement in lung function.
Wuytack (2018) [36]	N = 10 10 RCTs	934	Exercise-based HPR/Exercise-based community PR: strength training, LLE, ULE, aerobic training, walking, cycling, resistance training, muscle strengthening exercises	OPR	1) HRQoL: CRQ, SGRQ 2) Exercise capacity: 6MWD 3) Dyspnea: CRQ-D	Cochrane criteria	There was low to moderate evidence that outpatient and home-based exercise are equally effective.
Chen (2020) [37]	N = 9 9 RCTs	859	Exercise-based HPR	Center-based PR	1) HRQoL: SGRQ, CRQ 2) Exercise capacity: 6MWT, ESWT 3) Dyspnea: mMRC, CRQ-D	Cochrane criteria	Home and center-based pulmonary rehabilitation have similar effects on exercise capacity, quality of life, and dyspnoea scores in individuals with chronic obstructive pulmonary disease.
Fu (2021) [38]	N = 23 23 RCTs	2352	Exercise-based HPR: endurance training, resistance/strength training, walking, gymnastics, jogging, cycling, Tai Chi, Ba Duan Jin, stair climbing	Standard medical care	1) HRQoL: SGRQ 2) Exercise capacity: 6MWT 3) Pulmonary function: FEV_1_, FVC, FEV_1_/FVC	Jadad	Community-based pulmonary rehabilitation for stable COPD patients can significantly improve their lung function and quality of life.
Mendes Xavier (2022) [39]	N = 17 17 RCTs	898	Exercise-based HPR: LLE, ULE, walking, climbing, cycling, stretching, relaxation exercises, physical training, climbing up and down a ladder, resistance training, strengthening exercises, endurance training	Conventional PR	1) HRQoL: SGRQ 2) Exercise capacity: 6MWT 3) Dyspnea: CRQ-D, MRC, mMRC	Cochrane criteria	Home pulmonary rehabilitation reduced dyspnea levels, increased 6MWD, and improved HRQoL in COPD patients.
Paixão (2022) [40]	N = 11 10 RCTs 1 non-RCT	1205	Exercise-based HPR: stair-climbing, LLE, ULE, walking, endurance training, HIIT, strength training, resistance training	Usual care	1) HRQoL: SGRQ, CRQ 2) Exercise capacity: 6MWD, ISWD 3) Dyspnea: CRQ-D	Quality Assessment Tool for Quantitative Studies	Unsupervised physical activity interventions benefit dyspnea and exercise capacity of people with COPD, are safe, and present a high adherence rate.

Abbreviations: RCTs: randomized controlled trials; HPR: home pulmonary rehabilitation; LLE: lower-limb endurance exercise training; PR: pulmonary rehabilitation; HRQoL: health-related quality of life; CRQ: chronic respiratory disease questionnaire; SGRQ: St George’s respiratory questionnaire; 6MWD: 6-minute walk distance; SWT: shuttle walk test; MRC: medical research council; Borg: Borg scale; PEDro: physiotherapy evidence database; ULE: upper-limb endurance exercise training; CRQ-D: the CRQ domains of dyspnea; FEV1: forced expiratory volume in 1 second; FVC: forced volume vital capacity; OPR: outpatient pulmonary rehabilitation; ISWT: incremental shuttle walk test; EWST: endurance shuttle walk test; mMRC: modified British Medical Research Council; Jadad: Jadad scale; HIIT: high-intensity interval training; ISWD: incremental shuttle walk distance.

### 3.3. Overlap of reviews

A total of 11 SRs/MAs were included in this study, and the number of all original studies involved was 165 and 86 after deduplication. According to the formula CCA = (165–86) / (11×86–86) = 0.092, there is a slight overlap. The overlap matrix is shown in S3 Table.

### 3.4. Methodological quality

Table 2 shows the methodological quality assessment results of the included reviews. Of the 11 SRs/MAs, only one systematic review [16] was judged to be of low quality, and the quality of the other studies was assessed as very low. The AMSTAR 2 tool’s emphasis items are 2, 4, 7, 9, 11, 13, and 15. For item 2, four studies [16, 36, 39, 40] offered a protocol registration or publication before commencement. For item 4, only four included studies [16, 36, 39, 40] showed the adoption of a specific search strategy. Regarding item 7, only one study [36] supplied a list of eliminated documents and the reasons for their exclusion. For item 9, ten reviews [16, 31, 33–40] evaluated the risk of bias in every study with appropriate tools. For item 11, nine [16, 33–40] analyzed the data statistically using relevant procedures, and one study [31] used narrative SR without quantitative analysis. For item 13, only one study [16] considered the risk of bias when presenting the findings. Regarding item 15, two studies [16, 38] considered publication bias in interpreting or discussing the results. Furthermore, none of the researchers stated why the study was chosen nor indicated the funding sources for the included study.

**Table 2:** Result of the AMSTAR-2 assessments.

First Author, year	Q1	Q2	Q3	Q4	Q5	Q6	Q7	Q8	Q9	Q10	Q11	Q12	Q13	Q14	Q15	Q16	Ranking of quality
Vieira (2010) [31]	Y	N	N	PY	Y	Y	PY	Y	Y	N	NC	NC	N	N	NC	Y	critically low
Wang (2013) [32]	Y	N	N	PY	N	N	N	Y	N	N	N	N	N	N	N	N	critically low
Liu (2014) [33]	Y	N	N	PY	Y	Y	N	Y	Y	N	Y	Y	N	N	N	PY	critically low
Liu (2016) [34]	Y	N	N	PY	Y	Y	N	N	Y	N	Y	N	N	N	N	PY	critically low
Neves (2016) [16]	Y	Y	N	Y	Y	Y	N	Y	Y	N	Y	Y	Y	Y	Y	N	low
Li (2017) [35]	Y	N	N	PY	Y	N	N	Y	Y	N	Y	Y	N	PY	N	Y	critically low
Wuytack (2018) [36]	Y	Y	N	Y	Y	Y	Y	Y	Y	N	Y	Y	N	N	N	N	critically low
Chen (2020) [37]	Y	N	N	PY	N	N	N	Y	Y	N	Y	N	N	N	N	Y	critically low
Fu (2021) [38]	Y	N	N	PY	Y	N	N	Y	Y	N	Y	Y	N	N	Y	N	critically low
Mendes Xavier (2022) [39]	Y	Y	N	Y	Y	Y	N	Y	Y	N	Y	Y	N	Y	N	Y	critically low
Paixão (2022) [40]	Y	Y	N	Y	Y	Y	N	Y	Y	N	Y	N	N	N	N	Y	critically low

Abbreviations: Y: yes; PY: partial yes; N: no; NC: not conducted.

### 3.5. Report quality

**Fig 2:**
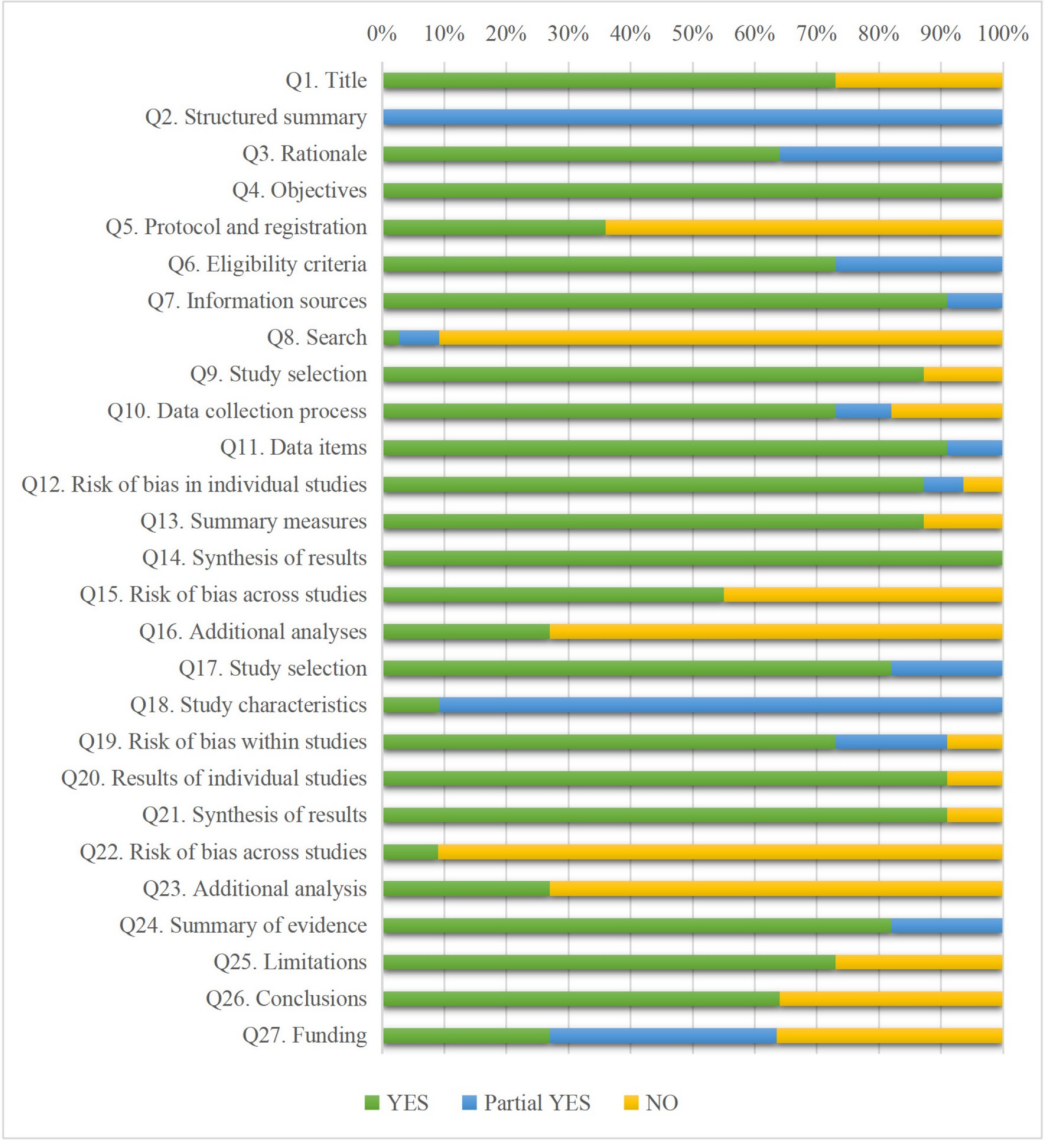
PRISMA score results for each item.

The percentage of included studies that met each of the 27 PRISMA criteria for transparent reporting is shown in Fig 2. Seventeen out of 27 items were adequately reported, over 70%. The objectives and synthesis of results were reported adequately (100%). The abstract was written inadequately (0%). In the section on methods, Q5 (protocol and registration), Q8 (search), and Q16 (additional analyses) reported incomplete (≤50%); Q22 (risk of bias across studies), and Q23 (additional analyses) in the results section were not adequately described (≤50%); insufficient detail in the description of Q27 (funding) (27%). Overall, two SRs/MAs [16, 39] reached over 85% compliance. The specific evaluation content is shown in S4 Table.

### 3.6. Quality of evidence for relevant outcomes

The quality of evidence for all results varies from very low to moderate. No outcome was categorized as high quality. Vieira’s study [31] had only qualitative analysis results and could not be assessed for the quality of evidence. Ten SRs/MAs [16, 32–40] included 71 outcomes. Of these outcome indicators, 12 were of moderate quality, 17 were of low quality, and 38 were of very low quality. The main causes for demotion were the limitations of the original research, followed by imprecision, publication bias, and inconsistency. S5 Table shows the GRADE evaluation in detail.

### 3.7. Outcomes

The proportion of results recorded in each of the eleven SRs/MAs is shown below: HRQoL (11/11, 100%), exercise capacity (11/11, 100%), dyspnea (10/11, 91%), and pulmonary function (3/11, 27%).

### 3.8. Effects of interventions

Tables 3 and 4 show the assessment of outcomes in the included studies.

**Table 3:** HPR versus control groups.

Outcomes	Tools	First Author
HRQoL	CRQ	Wang [32], Liu [33], Liu [34], Neves [16], Li [35]
	SGRQ	Liu [33], Liu [34], Neves [16], Li [35], Fu [38]
	Descriptive synthesis	Vieira [31], Paixão [40]
Exercise capacity	6MWT/6MWD	Wang [32], Liu [33], Neves [16], Li [35], Fu [38], Paixão [40]
	ISWT/ISWD	Neves [16], Paixão [40]
	SWT	Li [35]
	Descriptive synthesis	Vieira [31], Liu [34]
Dyspnea	Borg score	Liu [33], Liu [34], Li [35]
	CRQ-dyspnea	Wang [32], Liu [33], Liu [34], Neves [16], Li [35], Paixão [40]
	MRC	Neves [16], Li [35]
	Descriptive synthesis	Vieira [31]
Pulmonary function	-	Liu [33], Li [35], Fu [38]

**Table 4:** HPR versus OPR.

Outcomes	Tools	First Author
HRQoL	CRQ	Neves [16], Wuytack [36], Chen [37]
	SGRQ	Wuytack [36], Chen [37], Mendes Xavier [39]
	Descriptive synthesis	Vieira [31]
Exercise capacity	6MWT/6MWD	Neves [16], Wuytack [36], Chen [37], Mendes Xavier [39]
	EWST	Chen [37]
	Descriptive synthesis	Vieira [31]
Dyspnea	CRQ-dyspnea	Neves [16], Wuytack [36], Chen [37], Mendes Xavier [39]
	MRC/mMRC	Chen [37], Mendes Xavier [39]
	Descriptive synthesis	Vieira [31]

#### 3.8.1. HPR versus control groups (usual care, standard medical care, or no intervention)

1. HRQoL
  Eight reviews [16, 31–35, 38, 40] reported that exercise-based HPR has the potential to improve overall HRQoL compared to control groups. The strongest evidence (moderate) comes from Neves [16], which describes that exercise-based HPR improved the CRQ fatigue scores of subjects in the intervention group. Five included studies [16, 32–35] used the total score of CRQ to assess HRQoL after intervention. The results showed that the CRQ total scores of COPD patients in the HPR group were significantly higher than that in the control group, and the difference was statistically significant. The analysis results of the five included studies [16, 33–35, 38] showed that after the intervention, the SGRQ score of the HPR group was significantly improved, and the HRQoL was improved.
2. Exercise capacity
  Eight studies [16, 31–35, 38, 40] evaluated the effect of HPR on exercise ability. Six studies [16, 32, 33, 35, 38, 40] showed that 6MWT/6MWD in the HPR group was significantly improved compared with the control group, and the difference was statistically significant. Two studies [16, 40] used ISWT/ISWD to assess exercise capacity and found enhanced exercise capacity in the HPR group compared to the control group. Furthermore, one study [35] used SWT to test the exercise capacity of the two groups, and a meta-analysis showed that the difference was not statistically significant.
3. Dyspnea
  The effects of HPR on dyspnea were reported in seven studies [16, 31–35, 40]. In three reviews on measuring dyspnea with Borg score [33–35], meta-analysis results showed that after intervention, the dyspnea score of the HPR group was lower than that of the control group. The medium-quality study [40] found that the CRQ Dyspnea score of the HPR group had statistically significant improvement, but there was no clinically significant improvement. According to the evidence from four SRs/MAs [16, 32–34], participants who received the intervention exhibited considerable improvement assessed by CRQ-Dyspnea. The study with the highest quality [16] used MRC to evaluate the dyspnea of the two groups of subjects. The results showed that although the MRC score of the HPR group was significantly lower than that of the control group after the intervention, the difference was not statistically significant.
4. Pulmonary function
  Three reviews [33, 35, 38] reported the pulmonary function of the two groups of subjects after intervention. Liu’s study [33] indicated that HPR seems to have some sound effects on increasing pulmonary function in COPD patients, according to FEV1/FVC assessments. Li’s MAs [35] showed that HPR is ineffective in increasing pulmonary function in individuals with stable COPD. According to Fu’s MAs [38], compared with the control group, the main indicators of pulmonary function of stable COPD patients receiving exercise-based HPR treatment have significantly improved, with statistically significant differences.

#### 3.8.2. HPR versus OPR

1. HRQoL
  According to five reviews, there was no variation in HRQoL between HPR and outpatient PR [16, 31, 36, 37, 39]. There was no significant difference between the two interventions regarding CRQ scores for each domain [16, 36, 37]. OPR and HPR were equally effective in improving SGRQ scores [36, 37, 39].
2. Exercise capacity
  Five reviews [16, 31, 36, 37, 39] reported that exercise-based HPR has similar effects on exercise capacity compared to OPR. Moderate quality evidence [39] showed no significant difference in the 6MWT/6MWD data between HPR and OPR. One study [37] reported similar EWST results between exercise-based HPR and center-based PR.
3. Dyspnea
  According to the meta-analysis findings, there is no difference between exercise-based home or community pulmonary rehabilitation and OPR [16, 31, 36, 37, 39]. A comparison of HPR and OPR showed no difference in CRQ-dyspnea scores [16, 36, 37, 39]. The meta-analysis did not find a difference in mMRC scores between the HPR and OPR groups [37, 39].

## 4. Discussion

This overview of systematic reviews was intended to summarize the key features and evaluate the quality of evidence from selected SRs/MAs about the efficacy of exercise-based HPR in COPD.

### 4.1. Evidence quality summary

Although almost all included SRs/MAs have come to positive conclusions that there is substantial evidence that HPR is helpful, the quality of the evidence is inadequate to draw strong judgments. The overall methodological and statistical presentation quality of these included studies were typically low, according to the AMSTAR-2, PRISMA, and GRADE appraisal results.

This overview applied AMSTAR-2 to evaluate the methodological quality of selected SRs/MAs. Most researchers lacked a clear presentation and explanation of preregistration study processes, thorough search methodologies, and reasons for excluding literature. This could undermine the transparency of the studies that were included, as well as the dependability of the results. None of the 11 SRs/MAs indicated why certain types of studies were included, making it difficult to guarantee that reasonable inclusion criteria were met. The source of funding of original studies was not reported by all eleven reviews, which may affect the reliability due to potential conflicts of interest. In addition, the included SRs/MAs have flaws such as the risk of bias, heterogeneity, and publication bias, all of which impair the validity of the evidence quality. Except for one study whose methodological quality was low, most of the included reviews were extremely low, according to AMSTAR-2. The above problems should be addressed in future research.

The included reviews were of average quality, according to the PRISMA tool. The structured summary, protocol and registration, search strategy, additional analyses, risk of bias across studies, and funding details were standard low-scoring criteria. The results showed that items 4, 7, 9, 11–14, 17–18, 20–21, and 24 provided sufficient descriptions, but the rest received poor ratings. As a result, future research should adhere to PRISMA guidelines.

This study used the GRADE evaluation tool to rate the level of evidence for the outcome indicators included in the literature, with most indicators having a very low to moderate level of evidence. The main reason for the downgrading of the evaluation was study limitations, mainly in the implementation of randomization, blinding, and allocation concealment schemes in the original study were imperfect. This may be due to the characteristics of the intervention, which makes it difficult to achieve blinding of patients and investigators, and future clinical studies should further improve their methodological quality. The second is the large heterogeneity among the original studies, which directly reduces the reliability of the evidence. This needs to be addressed with further clarification of inclusion and exclusion criteria and appropriate subgroup analysis. Meanwhile, some included literature had more serious problems of imprecision and publication bias. Some Chinese literature included a small sample of studies, and some studies were not analyzed for publication bias, with some risk of publication bias. All of the above factors can lead to discrepancies between study findings and the actual situation, which means that more high-quality randomized controlled studies are needed to provide reliable data.

### 4.2. Summary of major discoveries

Most of the included studies [16, 31–35, 38, 40] suggested that HPR improves HRQoL and exercise capacity and relieves dyspnea symptoms compared to COPD patients who had usual care or regular medical treatment. COPD patients may experience peripheral muscle dysfunction, dyspnea, decreased exercise tolerance, and a lower HRQoL [41]. Changes in daily life activities in COPD patients correspond to improvements in HRQoL. The positive changes in these outcome indicators may be because that HPR increases the skeletal muscle strength of patients and can sustain change in their health behaviors [33]. Additionally, none of the incorporated SRs/MAs recorded adverse events in patients receiving exercise-based HPR, which appears safe for COPD patients. However, we cannot confirm whether HPR significantly affects pulmonary function, possibly because only three studies [33, 35, 38] included pulmonary function metrics. Further research into the effect of HPR intervention on pulmonary function in COPD patients is still needed.

Compared to OPR, HPR does not have worse outcomes. In terms of improving HRQoL, exercise capacity, and relieving symptoms of dyspnea, these studies [16, 31, 36, 37, 39] confirmed that HPR and OPR have similar effects. The main drawback of OPR is its limited availability. Patient-related barriers include travel and transportation to rehabilitation centers, inconvenient hours, weather factors, illness, and disruptions to established routines [42]. A qualitative study found that participants were more comfortable with pulmonary rehabilitation in a home setting than in a hospital or center [43]. Most participants felt that HPR was able to overcome the barriers of OPR while gaining support from groups such as friends, family, and neighbors. Therefore, HPR is more flexible and convenient, making it a viable alternative for some patients who are unwilling or unable to participate in hospital or outpatient rehabilitation programs.

### 4.3. Suggestions for future research

Although HPR may have short-term efficacy in COPD patients, the current analysis found no evidence of long-term efficacy of exercise-based HPR. We recommend that future RCTs be designed with extended follow-up to determine the long-term clinical effectiveness of HPR. At the same time, to minimize bias as much as feasible, we recommend that subgroup analysis of SR/MA adhere to a consistent intervention, duration and follow-up, and outcome assessments. HPR as an alternative format, appears to be safe and feasible, but further research should be done to determine the effects of home-based pulmonary rehabilitation on other outcomes.

HPR is less expensive in terms of time and space but has similar costs to OPR in terms of professional guidance and medical examinations [44]. It has not been possible to determine the economic differences between the two [45]. Future studies should also consider the financial costs of home pulmonary rehabilitation, including health services and individual costs, which require a comprehensive economic analysis of the costs and benefits to patients and health systems.

We recommend that future trials be adequately randomized and concealed, with the blinding of outcome assessors and statistical analysts. Minimized knowledge of which patients received the trial intervention and which patients received the control intervention in the clinical study. The monitoring mechanism is also improved so that the study designer is not involved in the implementation of the trial and does not have direct contact with patients, thus reducing the impact of bias on the study. It is also recommended that researchers report as completely as possible in the blinded and allocation concealment implementation of the study.

### 4.4. Limitations

First, there may be some duplicate original papers in the enrolled reviews. Although we briefly describe the overlap of articles in the included SRs/MAs, we did not explore these overlaps systematically. As a result, this may lead to inaccurate data reporting. Second, the AMSTAR-2, PRISMA, and GRADE assessment is a subjective process. There is no guarantee of the accuracy of the assessor’s assessment. Third, we only focused on a few outcomes, which may not reflect HPR’s combined efficacy in COPD. Fourth, due to linguistic constraints, we included SRs/MAs covering only Chinese and English literature, and the search may not have been comprehensive. In addition, we recommend that the monitoring mechanisms be improved so that researchers report as completely as possible in the blinded and allocation concealment implementation of the study.

## 5. Conclusions

This overview of SRs/MAs suggests that exercise-based HPR may positively affect COPD. Moreover, these meta-analysis results show that exercise-based HPR is not worse than outpatient/center-based PR, and from this standpoint, it may be an alternative to maintaining outpatient/center-based PR. However, these conclusions were restricted by the methodology, reporting quality, and evidence quality for all included SRs/MAs.

## Supporting information

S1 Table Search strategy. (DOCX)

S2 Table Exclude articles. (DOCX)

S3 Table Overlap matrix. (DOCX)

S4 Table Reporting quality assessment of systematic reviews by PRISMA. (DOCX)

S5 Table Results of evidence quality (based on the GRADE tool). (DOCX)

S6 Table Prisma 2009 checklist. (DOCX)

S7 Table AMSTAR-2 domains. (DOCX)
