# Supplementary material for: Effects of exercise-based home pulmonary rehabilitation on patients with chronic obstructive pulmonary disease: An overview of systematic review
Source: PLoS One. 2022 Nov 17;17(11):e0277632. doi: 10.1371/journal.pone.0277632 (PMC9671331; doi:10.1371/journal.pone.0277632)
Supplement: S1 Table — (DOCX) [file pone.0277632.s001.docx]

**Supplementary Table 1. Search strategy**

**(1)** **PUBMED**

| **Search** | **Query** |
| --- | --- |
| #1 | ((((((((((((((("Exercise"[Mesh]) OR ("Walking"[Mesh]) OR "Running"[Mesh]) OR "Swimming"[Mesh]) OR "Dancing"[Mesh]) OR "Yoga"[Mesh]) OR "Exercise Movement Techniques"[Mesh]) OR "Gymnastics"[Mesh]) OR "Exergaming"[Mesh]) OR "Muscle Stretching Exercises"[Mesh]) OR "Circuit-Based Exercise"[Mesh]) OR "Endurance Training"[Mesh]) OR "High-Intensity Interval Training"[Mesh]) OR "Plyometric Exercise"[Mesh]) OR "Resistance Training"[Mesh]) OR "Stair Climbing"[Mesh]) OR "Warm-Up Exercise"[Mesh] |
| #2 | (((((((((((((((((((((((((((((((((((("exercise"[Title/Abstract]) OR ("exercises"[Title/Abstract])) OR ("training"[Title/Abstract])) OR ("exercise training"[Title/Abstract])) OR ("physical activity"[Title/Abstract])) OR ("physical exercise"[Title/Abstract])) OR ("exercise program"[Title/Abstract])) OR ("aerobic training"[Title/Abstract])) OR ("walking"[Title/Abstract])) OR ("ambulation"[Title/Abstract])) OR ("running"[Title/Abstract])) OR ("swimming"[Title/Abstract])) OR ("jogging"[Title/Abstract])) OR ("activities of daily living"[Title/Abstract])) OR ("dancing"[Title/Abstract])) OR ("yoga"[Title/Abstract])) OR ("exercise movement techniques"[Title/Abstract])) OR ("pilates"[Title/Abstract])) OR ("tai-chi"[Title/Abstract])) OR ("qigong"[Title/Abstract])) OR ("tai ji"[Title/Abstract])) OR ("whole-body vibration"[Title/Abstract])) OR ("pedometer"[Title/Abstract])) OR ("pedometer-based"[Title/Abstract])) OR ("cool-down exercise"[Title/Abstract])) OR ("exergaming"[Title/Abstract])) OR ("gymnastics"[Title/Abstract])) OR ("muscle stretching exercises"[Title/Abstract])) OR ("circuit-based exercise"[Title/Abstract])) OR ("endurance training"[Title/Abstract])) OR ("high-intensity interval training"[Title/Abstract])) OR ("HIIT"[Title/Abstract])) OR ("plyometric exercise"[Title/Abstract])) OR ("resistance training"[Title/Abstract])) OR ("stair climbing"[Title/Abstract])) OR ("warm-up exercise"[Title/Abstract])) OR ("acute exercise"[Title/Abstract]) |
| #3 | ("pulmonary rehabilitation"[Title/Abstract]) OR ("PR"[Title/Abstract]) |
| #4 | #1 OR #2 OR #3 |
| #5 | "Telerehabilitation"[Mesh] |
| #6 | ((((((((((("homebase"[Title/Abstract]) OR ("home"[Title/Abstract])) OR ("community"[Title/Abstract])) OR ("home-based"[Title/Abstract])) OR ("outside the hospital"[Title/Abstract])) OR ("out of hospital"[Title/Abstract])) OR ("telerehabilitation"[Title/Abstract])) OR ("tele rehabilitation"[Title/Abstract])) OR ("tele-rehab"[Title/Abstract])) OR ("tele-rehabilitation"[Title/Abstract])) OR ("tele-r"[Title/Abstract])) OR ("remote rehabilitation"[Title/Abstract]) |
| #7 | #5 OR #6 |
| #8 | "Pulmonary Disease, Chronic Obstructive"[Mesh] |
| #9 | ((((((("COPD"[Title/Abstract]) OR ("chronic obstructive pulmonary disease"[Title/Abstract])) OR ("chronic obstructive lung disease"[Title/Abstract])) OR ("chronic obstructive airway disease"[Title/Abstract])) OR ("chronic obstructive lung disease"[Title/Abstract])) OR ("coad"[Title/Abstract])) OR ("chronic obstructive airway disease"[Title/Abstract])) OR ("chronic airflow obstruction"[Title/Abstract]) |
| #10 | #8 OR #9 |
| #11 | ("Systematic Review" [Publication Type]) OR ("Meta-Analysis" [Publication Type]) OR "Meta-Analysis as Topic"[Mesh] OR "Systematic Reviews as Topic"[Mesh] |
| #12 | (((((("systematic review"[Title/Abstract]) OR ("meta analysis"[Title/Abstract])) OR ("meta analyses"[Title/Abstract])) OR ("meta-analysis"[Title/Abstract])) OR ("meta-analyses"[Title/Abstract])) OR ("metaanalysis"[Title/Abstract])) OR ("metaanalyses"[Title/Abstract]) |
| #13 | #11 OR #12 |
| #14 | #4 AND #7 AND #10 AND #13 |

**(2) Web of Science**

| **Search** | **Query** |
| --- | --- |
| #1 | TS=(“exercise” OR “exercises” OR “training” OR “exercise training” OR “physical activity” OR “physical exercise” OR “exercise program” OR “aerobic training” OR “walking” OR “ambulation” OR “running” OR “swimming” OR “jogging” OR “activities of daily living” OR “dancing” OR “yoga” OR “exercise movement techniques” OR “pilates” OR “tai-chi” OR “qigong” OR “tai ji” OR “whole-body vibration” OR “pedometer” OR “pedometer-based” OR “cool-down exercise” OR “exergaming” OR “gymnastics” OR “muscle stretching exercises” OR “circuit-based exercise” OR “endurance training” OR “high-intensity interval training” OR “HIIT” OR “plyometric exercise” OR “resistance training” OR “stair climbing” OR “warm-up exercise” OR “acute exercise”) |
| #2 | TS=(“pulmonary rehabilitation” OR “PR”) |
| #3 | #1 OR #2 |
| #4 | TS=(“homebase” OR “home” OR “community” OR “home-based” OR “outside the hospital” OR “out of hospital” OR “telerehabilitation” OR “tele rehabilitation” OR “tele-rehab” OR “tele-rehabilitation” OR “tele-r” OR “remote rehabilitation”) |
| #5 | TS=(“COPD” OR “chronic obstructive pulmonary disease” OR “chronic obstructive lung disease” OR “chronic obstructive airway disease” OR “chronic obstructive lung disease” OR “coad” OR “chronic obstructive airway disease” OR “chronic airflow obstruction”) |
| #6 | TS=(“systematic review” OR “meta analysis” OR “meta analyses” OR “meta-analysis” OR “meta-analyses” OR “metaanalysis” OR “metaanalyses”) |
| #7 | #3 AND #4 AND #5 AND #6 |

**(3) Cochrane library**

| **Search** | **Query** |
| --- | --- |
| #1 | MeSH descriptor: [Exercise] explode all trees |
| #2 | MeSH descriptor: [Walking] explode all trees |
| #3 | MeSH descriptor: [Running] explode all trees |
| #4 | MeSH descriptor: [Swimming] explode all trees |
| #5 | MeSH descriptor: [Dancing] explode all trees |
| #6 | MeSH descriptor: [Yoga] explode all trees |
| #7 | MeSH descriptor: [Exercise Movement Techniques] explode all trees |
| #8 | MeSH descriptor: [Gymnastics] explode all trees |
| #9 | MeSH descriptor: [Exergaming] explode all trees |
| #10 | MeSH descriptor: [Muscle Stretching Exercises] explode all trees |
| #11 | MeSH descriptor: [Circuit-Based Exercise] explode all trees |
| #12 | MeSH descriptor: [Endurance Training] explode all trees |
| #13 | MeSH descriptor: [High-Intensity Interval Training] explode all trees |
| #14 | MeSH descriptor: [Plyometric Exercise] explode all trees |
| #15 | MeSH descriptor: [Resistance Training] explode all trees |
| #16 | MeSH descriptor: [Stair Climbing] explode all trees |
| #17 | MeSH descriptor: [Warm-Up Exercise] explode all trees |
| #18 | # 1 OR #2 OR #3 OR #4 OR #5 OR #6 OR #7 OR #8 OR #9 OR #10 OR #11 OR #12 OR #13 OR #14 OR #15 OR #16 OR #17 |
| #19 | (“exercise” OR “exercises” OR “training” OR “exercise training” OR “physical activity” OR “physical exercise” OR “exercise program” OR “aerobic training” OR “walking” OR “ambulation” OR “running” OR “swimming” OR “jogging” OR “wounds” OR “activities of daily living” OR “dancing” OR “yoga” OR “exercise movement techniques” OR “pilates” OR “tai-chi” OR “qigong” OR “tai ji” OR “whole-body vibration” OR “pedometer” OR “pedometer-based” OR “cool-down exercise” OR “exergaming” OR “gymnastics” OR “muscle stretching exercises” OR “circuit-based exercise” OR “endurance training” OR “high-intensity interval training” OR “HIIT” OR “plyometric exercise” OR “resistance training” OR “stair climbing” OR “warm-up exercise” OR “acute exercise”):ti,ab,kw |
| #20 | (“pulmonary rehabilitation” OR “PR”):ti,ab,kw |
| #21 | #18 OR #19 OR #20 |
| #22 | MeSH descriptor: [Telerehabilitation] explode all trees |
| #23 | (“homebase” OR “home” OR “community” OR “home-based” OR “outside the hospital” OR “out of hospital” OR “telerehabilitation” OR “tele rehabilitation” OR “tele-rehab” OR “tele-rehabilitation” OR “tele-r” OR “remote rehabilitation”):ti,ab,kw |
| #24 | #22 OR #23 |
| #25 | MeSH descriptor: [Pulmonary Disease, Chronic Obstructive] explode all trees |
| #26 | (“COPD” OR “chronic obstructive pulmonary disease” OR “chronic obstructive lung disease” OR “chronic obstructive airway disease” OR “chronic obstructive lung disease” OR “coad” OR “chronic obstructive airway disease” OR “chronic airflow obstruction”):ti,ab,kw |
| #27 | #25 OR #26 |
| #28 | MeSH descriptor: [Meta-Analysis as Topic] explode all trees |
| #29 | MeSH descriptor: [Systematic Reviews as Topic] explode all trees |
| #30 | #28 OR #29 |
| #31 | (“systematic review” OR “meta analysis” OR “meta analyses” OR “meta-analysis” OR “meta-analyses” OR “metaanalysis” OR “metaanalyses”):ti,ab,kw |
| #32 | #30 OR #31 |
| #33 | #21 AND #24 AND #27 AND #32 |

**(4) EMBASE**

| **Search** | **Query** |
| --- | --- |
| #1 | 'exercise'/exp OR 'walking'/exp OR 'running'/exp OR 'swimming'/exp OR 'dancing'/exp OR 'yoga'/exp OR 'kinesiotherapy'/exp OR 'gymnastics'/exp OR 'exergaming'/exp OR 'stretching exercise'/exp OR 'circuit training'/exp OR 'endurance training'/exp OR 'high intensity interval training'/exp OR 'resistance training'/exp OR 'stair climbing'/exp |
| #2 | 'exercise':ab,ti OR 'exercises':ab,ti OR 'training':ab,ti OR 'exercise training':ab,ti OR 'physical activity':ab,ti OR 'physical exercise':ab,ti OR 'exercise program':ab,ti OR 'aerobic training':ab,ti OR 'walking':ab,ti OR 'ambulation':ab,ti OR 'running':ab,ti OR 'swimming':ab,ti OR 'jogging':ab,ti OR 'activities of daily living':ab,ti OR 'dancing':ab,ti OR 'yoga':ab,ti OR 'exercise movement techniques':ab,ti OR 'pilates':ab,ti OR 'tai-chi':ab,ti OR 'qigong':ab,ti OR 'tai ji':ab,ti OR 'whole-body vibration':ab,ti OR 'pedometer':ab,ti OR 'pedometer-based':ab,ti OR 'cool-down exercise':ab,ti OR 'exergaming':ab,ti OR 'gymnastics':ab,ti OR 'muscle stretching exercises':ab,ti OR 'circuit-based exercise':ab,ti OR 'endurance training':ab,ti OR 'high-intensity interval training':ab,ti OR 'HIIT':ab,ti OR 'plyometric exercise':ab,ti OR 'resistance training':ab,ti OR 'stair climbing':ab,ti OR 'warm-up exercise':ab,ti OR 'acute exercise':ab,ti |
| #3 | 'pulmonary rehabilitation':ab,ti OR 'PR':ab,ti |
| #4 | #1 OR #2 OR #3 |
| #5 | 'telerehabilitation'/exp OR 'home'/exp OR 'community'/exp |
| #6 | 'homebase':ab,ti OR 'home':ab,ti OR 'community':ab,ti OR 'home-based':ab,ti OR 'outside the hospital':ab,ti OR 'out of hospital':ab,ti OR 'telerehabilitation':ab,ti OR 'tele rehabilitation':ab,ti OR 'tele-rehab':ab,ti OR 'tele-rehabilitation':ab,ti OR 'tele-r':ab,ti OR 'remote rehabilitation':ab,ti |
| #7 | #5 OR #6 |
| #8 | 'chronic obstructive lung disease'/exp |
| #9 | 'COPD':ab,ti OR 'chronic obstructive pulmonary disease':ab,ti OR 'chronic obstructive lung disease':ab,ti OR 'chronic obstructive airway disease':ab,ti OR 'chronic obstructive lung disease':ab,ti OR 'coad ':ab,ti OR 'chronic obstructive airway disease':ab,ti OR 'chronic airflow obstruction':ab,ti |
| #10 | #8 OR #9 |
| #11 | 'systematic review'/exp OR 'meta analysis'/exp |
| #12 | 'systematic review':ab,ti OR 'meta analysis':ab,ti OR 'meta analyses':ab,ti OR 'meta-analysis':ab,ti OR 'meta-analyses':ab,ti OR 'metaanalysis':ab,ti OR 'metaanalyses ':ab,ti |
| #13 | #11 OR #12 |
| #14 | #4 AND #7 AND #10 AND #13 |

**(5) CNKI**

(TKA='家庭' or TKA='社区' or TKA='出院' or TKA='远程') and (TKA='肺康复' or TKA='锻炼' or TKA='训练' or TKA='运动' or TKA='太极' or TKA='有氧' or TKA='无氧' or TKA='体操' or TKA='游泳' or TKA='步行' or TKA='跑步' or TKA='跳舞') and (TKA='COPD' or TKA='慢阻肺' or TKA='慢性阻塞性肺疾病') and (TKA='Meta分析' or TKA='系统评价' or TKA='荟萃分析' or TKA='系统综述' or TKA='整合分析' or TKA='元分析')

**(6) Wanfang**

主题:("家庭" + "社区" + "出院" + "远程") and 主题:("肺康复" + "锻炼" + "训练" + "运动" + "太极" + "有氧" + "无氧" + "体操" + "游泳" + "步行" + "跑步" + "跳舞") and 主题:("COPD " + "慢阻肺" + "慢性阻塞性肺疾病") and 主题:(“Meta分析” + “系统评价” + “荟萃分析” + “系统综述” + “整合分析” + “元分析”)
