# Supplementary material for: Effects of exercise-based home pulmonary rehabilitation on patients with chronic obstructive pulmonary disease: An overview of systematic review
Source: PLoS One. 2022 Nov 17;17(11):e0277632. doi: 10.1371/journal.pone.0277632 (PMC9671331; doi:10.1371/journal.pone.0277632)
Supplement: S2 Table — (DOCX) [file pone.0277632.s002.docx]

**Supplementary Table 2. Exclude Articles**

| **First Author, year** | **Title** | **Reasons for exclusion** |
| --- | --- | --- |
| Wijkstra  2007 | Home based rehabilitation for patients with COPD: an attractive alternative. | Conference Abstract. |
| Pinto  2019 | Home-based pulmonary rehabilitation and follow-up strategies in COPD patients: A systematic review. |  |
| Hailey  2019 | Progress in use of telerehabilitation for persons with COPD. |  |
| Cox  2021 | Telerehabilitation for chronic respiratory disease: A Cochrane systematic review. |  |
| Barker  2020 | Home-based exercise training (HET) post-hospitalisation for acute exacerbation of COPD (AECOPD) - a mixed-method systematic review. |  |
| Lundell  2014 | Telehealthcare for patients with COPD, effects on physical activity level, physical capacity and dyspnea: A systematic review and meta-analysis |  |
| Arbillaga-Etxarri  2018 | Long-term efficacy and effectiveness of a behavioural and community-based exercise intervention (Urban Training) to increase physical activity in patients with COPD: a randomised controlled trial. | Not SR/MA. |
| Zhang  2013 | MIOTIC study: a prospective, multicenter, randomized study to evaluate the long-term efficacy of mobile phone-based Internet of Things in the management of patients with stable COPD. |  |
| Shi  2021 | Home-based versus outpatient pulmonary rehabilitation program for patients with chronic obstructive pulmonary disease: A protocol for systematic review and meta-analysis. | Protocol. |
| Uzzaman  2021 | Clinical effectiveness and components of Home-pulmonary rehabilitation for people with chronic respiratory diseases: A systematic review protocol. |  |
| Robinson  2018 | Facilitators and barriers to physical activity following pulmonary rehabilitation in COPD: a systematic review of qualitative studies. | Narrative systematic review. |
| Lahham  2018 | Home-based pulmonary rehabilitation for people with COPD: A qualitative study reporting the patient perspective. |  |
| Zhu  2017 | Effect of self-management program on health status for patients with Chronic Obstructive Pulmonary Disease: a meta-analysis | Interventions not based on exercise training. |
| Ding  2019 | Remote rehabilitation applied in patients with stable stage of chronic obstructive pulmonary disease: a meta-analysis. |  |
| Calvache-Mateo  2021 | Efficacy of Web-Based Supportive Interventions in Quality of Life in COPD Patients, a Systematic Review and Meta-Analysis. |  |
| Cruz  2014 | Home telemonitoring in COPD: A systematic review of methodologies and patients' adherence. |  |
| Hong  2019 | Effectiveness of tele-monitoring by patient severity and intervention type in chronic obstructive pulmonary disease patients: A systematic review and meta-analysis. |  |
| Bonnevie  2021 | Advanced telehealth technology improves home-based exercise therapy for people with stable chronic obstructive pulmonary disease: a systematic review. |  |
| Li  2020 | Telemonitoring Interventions in COPD Patients: Overview of Systematic Reviews. |  |
| Malaguti  2021 | Supervised maintenance programmes following pulmonary rehabilitation compared to usual care for chronic obstructive pulmonary disease. |  |
| Thomas  2010 | The impact of home-based physiotherapy interventions on breathlessness during activities of daily living in severe COPD: a systematic review. |  |
| Lundell  2015 | Telehealthcare in COPD: A systematic review and meta-analysis on physical outcomes and dyspnea. |  |
| Lu  2019 | Effects of Home-Based Breathing Exercises in Subjects With COPD. |  |
| Ora  2022 | Efficacy of respiratory tele-rehabilitation in COPD patients: Systematic review and meta-analysis. |  |
| Yin  2021 | Early application of endurance combined with resistance training after acute exacerbation of chronic obstructive pulmonary disease: a meta-analysis | Home-based pulmonary rehabilitation is a non-major intervention. |
| Gendron  2018 | Active mind‐body movement therapies as an adjunct to or in comparison with pulmonary rehabilitation for people with chronic obstructive pulmonary disease. |  |
| Higashimoto  2020 | Effect of pulmonary rehabilitation programs including lower limb endurance training on dyspnea in stable COPD: A systematic review and meta-analysis. |  |
| Jolly  2016 | Self-management of health care behaviors for COPD: a systematic review and meta-analysis. |  |
| Taylor  2021 | Efficacy of unsupervised exercise in adults with obstructive lung disease: a systematic review and meta-analysis. | Not only COPD patients. |
| Hanrahan  2021 | Behaviour change and physical activity interventions for physical activity engagement in community dwelling adults with chronic obstructive pulmonary disease: protocol for a systematic review. | Control group interventions did not meet the requirements. |
| Imamura  2020 | Long-term efficacy of pulmonary rehabilitation with home-based or low frequent maintenance programs in patients with chronic obstructive pulmonary disease: a meta-analysis. |  |
| Seixas  2016 | Home Rehabilitation with Unsupervised Exercise in Copd: A Systematic Review. |  |
| Ahn  2016 | A Systematic Review of Home based Pulmonary Rehabilitation in COPD Patients: Randomized Controlled Trials. | Non-Chinese and English papers. |
