# Supplementary material for: Effects of exercise-based home pulmonary rehabilitation on patients with chronic obstructive pulmonary disease: An overview of systematic review
Source: PLoS One. 2022 Nov 17;17(11):e0277632. doi: 10.1371/journal.pone.0277632 (PMC9671331; doi:10.1371/journal.pone.0277632)
Supplement: S3 Table — (DOCX) [file pone.0277632.s003.docx]

**Supplementary Table 3.** **Overlap matrix**

| **Overlap matrix** | **N1** | **N2** | **N3** | **N4** | **N5** | **N6** | **N7** | **N8** | **N9** | **N10** | **N11** | **NO.of times inclued** |
| --- | --- | --- | --- | --- | --- | --- | --- | --- | --- | --- | --- | --- |
| Fernández AM 2009 | Y | Y | Y | Y |  |  |  |  |  | Y |  | 5 |
| Maltais F 2008 | Y |  |  |  | Y |  | Y | Y |  |  |  | 4 |
| Strijbos JH 1996 | Y | Y |  |  |  |  | Y |  |  | Y |  | 4 |
| Güell MR 2008 | Y |  |  |  | Y |  | Y | Y |  |  |  | 4 |
| Puente-Maestu L 2000 | Y |  |  |  | Y |  | Y |  |  |  |  | 3 |
| Boxall AM 2005 | Y | Y | Y | Y |  | Y |  |  |  | Y |  | 6 |
| Busch AJ 1988 | Y | Y | Y |  |  |  |  |  |  |  |  | 3 |
| Hernández MT 2000 | Y |  | Y | Y | Y | Y |  |  |  | Y |  | 6 |
| McGavin CR 1977 | Y | Y |  |  |  |  |  |  |  |  | Y | 3 |
| Murphy N 2005 | Y | Y |  |  | Y |  |  |  |  | Y |  | 4 |
| Resqueti VR 2007 | Y | Y |  |  | Y | Y |  |  |  |  |  | 4 |
| Singh V 2003 | Y | Y | Y |  | Y | Y |  |  |  | Y |  | 6 |
| Wijkstra PJ 1994 |  | Y | Y |  | Y | Y |  |  |  |  |  | 4 |
| Wedzicha JA 1998 |  | Y | Y |  |  |  |  |  |  |  |  | 2 |
| XIE SL 2003 |  | Y | Y |  |  | Y |  |  |  |  |  | 3 |
| Oh EG 2003 |  | Y | Y | Y | Y | Y |  |  |  | Y |  | 6 |
| Moore J 2009 |  | Y |  |  | Y |  |  |  |  |  | Y | 3 |
| Wu 2009 |  | Y |  |  |  |  |  |  |  |  |  | 1 |
| Ghanem M 2010 |  | Y | Y | Y | Y |  |  |  |  | Y |  | 5 |
| Akinci AC 2011 |  |  | Y | Y | Y | Y |  |  |  | Y |  | 5 |
| du Moulin 2009 |  |  | Y | Y | Y |  |  |  |  |  |  | 3 |
| O'Shea SD 2007 |  |  | Y | Y |  | Y |  |  |  | Y |  | 4 |
| Koppers RJ 2006 |  |  | Y | Y |  |  |  |  |  | Y |  | 4 |
| Man WD 2004 |  |  | Y | Y | Y |  |  |  |  |  |  | 3 |
| Larson JL 1999 |  |  | Y |  |  |  |  |  |  |  |  | 1 |
| Wijkstra PJ 1996 |  |  | Y |  |  | Y |  |  |  |  |  | 2 |
| Bauldoff GS 1996 |  |  | Y |  |  |  |  |  |  |  |  | 1 |
| Bavarsad MB 2015 |  |  |  | Y |  | Y |  |  |  |  |  | 2 |
| Pradella CO 2015 |  |  |  | Y |  | Y |  |  |  |  |  | 2 |
| de Sousa Pinto JM 2014 |  |  |  | Y |  | Y |  |  |  | Y |  | 3 |
| Behnke M 2000 |  |  |  |  | Y |  |  |  |  |  |  | 1 |
| Cambach W 1997 |  |  |  |  | Y |  |  |  |  |  |  | 1 |
| Dias FD 2013 |  |  |  |  | Y |  |  |  |  |  |  | 1 |
| Effing T 2011 |  |  |  |  | Y |  |  |  |  |  |  | 1 |
| Elliott M 2004 |  |  |  |  | Y |  | Y |  |  |  |  | 2 |
| Ho CF 2012 |  |  |  |  | Y | Y |  |  |  |  | Y | 3 |
| Jang HJ 2006 |  |  |  |  | Y |  |  |  |  |  |  | 1 |
| Román M 2013 |  |  |  |  | Y |  |  |  |  |  |  | 1 |
| van Wetering CR 2010 |  |  |  |  | Y |  |  |  |  |  |  | 1 |
| Holland A 2016 |  |  |  |  |  |  | Y |  |  |  |  | 1 |
| Jolly E 2014 |  |  |  |  |  |  | Y |  |  |  |  | 1 |
| Mendes De Oliveira JC 2010 |  |  |  |  |  |  | Y | Y |  |  |  | 2 |
| Waterhouse JC 2010 |  |  |  |  |  |  | Y |  |  |  |  | 1 |
| Laval University 2005 |  |  |  |  |  |  | Y |  |  |  |  | 1 |
| Burge AT 2020 |  |  |  |  |  |  |  | Y |  |  |  | 1 |
| Chaplin E 2017 |  |  |  |  |  |  |  | Y |  |  |  | 1 |
| Bourne S 2017 |  |  |  |  |  |  |  | Y |  |  |  | 1 |
| Horton EJ 2017 |  |  |  |  |  |  |  | Y |  |  |  | 1 |
| Hansen H 2020 |  |  |  |  |  |  |  | Y |  |  |  | 1 |
| Holland AE 2017 |  |  |  |  |  |  |  | Y |  |  |  | 1 |
| LIAO OF 2011 |  |  |  |  |  |  |  |  | Y |  |  | 1 |
| SONG L 2012 |  |  |  |  |  |  |  |  | Y |  |  | 1 |
| HUANG XH 2012 |  |  |  |  |  |  |  |  | Y |  |  | 1 |
| XU JH 2013 |  |  |  |  |  |  |  |  | Y |  |  | 1 |
| LIANG ZQ 2013 |  |  |  |  |  |  |  |  | Y |  |  | 1 |
| LU ZS 2013 |  |  |  |  |  |  |  |  | Y |  |  | 1 |
| ZHENG RB 2014 |  |  |  |  |  |  |  |  | Y |  |  | 1 |
| WANG X 2014 |  |  |  |  |  |  |  |  | Y |  |  | 1 |
| GAO XF 2015 |  |  |  |  |  |  |  |  | Y |  |  | 1 |
| LI JN 2015 |  |  |  |  |  |  |  |  | Y |  |  | 1 |
| DONG YF 2015 |  |  |  |  |  |  |  |  | Y |  |  | 1 |
| QIU JF 2016 |  |  |  |  |  |  |  |  | Y |  |  | 1 |
| WANG L 2016 |  |  |  |  |  |  |  |  | Y |  |  | 1 |
| LI CN 2017 |  |  |  |  |  |  |  |  | Y |  |  | 1 |
| SHEN XL 2017 |  |  |  |  |  |  |  |  | Y |  |  | 1 |
| LI CQ 2017 |  |  |  |  |  |  |  |  | Y |  |  | 1 |
| QIAO L 2017 |  |  |  |  |  |  |  |  | Y |  |  | 1 |
| TAN JR 2018 |  |  |  |  |  |  |  |  | Y |  |  | 1 |
| TIAN SH 2018 |  |  |  |  |  |  |  |  | Y |  |  | 1 |
| LI SG 2019 |  |  |  |  |  |  |  |  | Y |  |  | 1 |
| JIA HZ 2020 |  |  |  |  |  |  |  |  | Y |  |  | 1 |
| GUO XH 2020 |  |  |  |  |  |  |  |  | Y |  |  | 1 |
| CUI KM 2020 |  |  |  |  |  |  |  |  | Y |  |  | 1 |
| Tabak M 2014 |  |  |  |  |  |  |  |  |  | Y |  | 1 |
| Tsai LLY 2017 |  |  |  |  |  |  |  |  |  | Y |  | 1 |
| Vasilopoulou M 2017 |  |  |  |  |  |  |  |  |  | Y |  | 1 |
| Jiang Y 2020 |  |  |  |  |  |  |  |  |  | Y |  | 1 |
| Ko FW 2021 |  |  |  |  |  |  |  |  |  | Y |  | 1 |
| Coultas DB 2018 |  |  |  |  |  |  |  |  |  |  | Y | 1 |
| Coultas DB 2016 |  |  |  |  |  |  |  |  |  |  | Y | 1 |
| Mitchell KE 2014 |  |  |  |  |  |  |  |  |  |  | Y | 1 |
| Chen Y 2018 |  |  |  |  |  |  |  |  |  |  | Y | 1 |
| Cameron-Tucker HL 2016 |  |  |  |  |  |  |  |  |  |  | Y | 1 |
| Elçi A 2008 |  |  |  |  |  |  |  |  |  |  | Y | 1 |
| Lin FL 2019 |  |  |  |  |  |  |  |  |  |  | Y | 1 |
| Lahham A 2020 |  |  |  |  |  |  |  |  |  |  | Y | 1 |
| **Total studies inclued** | **12** | **15** | **18** | **13** | **23** | **14** | **10** | **9** | **23** | **17** | **11** |  |

Notes: Marks (Y) indicate when a primary study is included in an SR/MA (Yes). N1=Vieira (2010) [31]; N2=Wang (2013) [32]; N3=Liu (2014) [33]; N4=Liu (2016) [34]; N5=Neves (2016) [16]; N6=

Li (2017) [35]; N7=Wuytack (2018) [36]; N8=Chen (2020) [37]; N9=Fu (2021) [38]; N10=Mendes Xavier (2022) [39]; N11=Paixão (2022) [40].
