# Supplementary material for: Effects of exercise-based home pulmonary rehabilitation on patients with chronic obstructive pulmonary disease: An overview of systematic review
Source: PLoS One. 2022 Nov 17;17(11):e0277632. doi: 10.1371/journal.pone.0277632 (PMC9671331; doi:10.1371/journal.pone.0277632)
Supplement: S4 Table — (DOCX) [file pone.0277632.s004.docx]

**Supplementary Table 4. Reporting quality assessment of systematic reviews by PRISMA**

| **Section/topic** | | **Vieira  (2010) [31]** | **Wang  (2013) [32]** | **Liu  (2014) [33]** | **Liu (2016) [34]** | **Neves  (2016) [16]** | **Li (2017) [35]** | **Wuytack**  **(2018) [36]** | **Chen  (2020) [37]** | **Fu (2021) [38]** | **Mendes Xavier  (2022) [39]** | **Paixão  (2022) [40]** | **Compliance (%)** |
| --- | --- | --- | --- | --- | --- | --- | --- | --- | --- | --- | --- | --- | --- |
| Title | Q1. Title | N | N | Y | N | Y | Y | Y | Y | Y | Y | Y | 73 |
| Abstract | Q2. Structured summary | PY | PY | PY | PY | PY | PY | PY | PY | PY | PY | PY | 0 |
| Introduction | Q3. Rationale | Y | PY | Y | PY | Y | PY | Y | Y | PY | Y | Y | 64 |
|  | Q4. Objectives | Y | Y | Y | Y | Y | Y | Y | Y | Y | Y | Y | 100 |
| Methods | Q5. Protocol and registration | N | N | N | N | Y | N | Y | N | N | Y | Y | 36 |
|  | Q6. Eligibility criteria | Y | Y | PY | PY | Y | Y | PY | Y | Y | Y | Y | 73 |
|  | Q7. Information sources | Y | PY | Y | Y | Y | Y | Y | Y | Y | Y | Y | 91 |
|  | Q8. Search | PY | PY | PY | PY | PY | PY | Y | PY | N | Y | Y | 27 |
|  | Q9. Study selection | Y | N | Y | Y | Y | Y | Y | N | Y | Y | Y | 82 |
|  | Q10. Data collection process | Y | N | Y | Y | Y | Y | Y | N | PY | Y | Y | 73 |
|  | Q11. Data items | Y | PY | Y | Y | Y | Y | Y | Y | Y | Y | Y | 91 |
|  | Q12. Risk of bias in individual studies | PY | N | Y | Y | Y | Y | Y | Y | Y | Y | Y | 82 |
|  | Q13. Summary measures | N | N | Y | Y | Y | Y | Y | Y | Y | Y | Y | 82 |
|  | Q14. Synthesis of results | Y | Y | Y | Y | Y | Y | Y | Y | Y | Y | Y | 100 |
|  | Q15. Risk of bias across studies | N | N | Y | N | Y | Y | Y | N | Y | Y | N | 55 |
|  | Q16. Additional analyses | N | N | N | N | Y | N | Y | N | N | Y | N | 27 |
| Results | Q17. Study selection | Y | PY | Y | PY | Y | Y | Y | Y | Y | Y | Y | 82 |
|  | Q18. Study characteristics | Y | Y | Y | PY | Y | Y | Y | Y | Y | Y | Y | 91 |
|  | Q19. Risk of bias within studies | Y | N | Y | PY | Y | Y | Y | Y | PY | Y | Y | 73 |
|  | Q20. Results of individual studies | N | Y | Y | Y | Y | Y | Y | Y | Y | Y | Y | 91 |
|  | Q21. Synthesis of results | N | Y | Y | Y | Y | Y | Y | Y | Y | Y | Y | 91 |
|  | Q22. Risk of bias across studies | N | N | N | N | Y | N | N | N | N | N | N | 9 |
|  | Q23. Additional analysis | N | N | N | N | Y | N | Y | N | N | Y | N | 27 |
| Discussion | Q24. Summary of evidence | Y | PY | Y | Y | Y | Y | Y | Y | PY | Y | Y | 82 |
|  | Q25. Limitations | Y | N | Y | N | Y | Y | N | Y | Y | Y | Y | 73 |
|  | Q26. Conclusions | Y | N | Y | N | Y | N | N | Y | Y | Y | Y | 64 |
| Funding | Q27. Funding | Y | N | PY | PY | N | PY | N | PY | N | Y | Y | 27 |
| Compliance (%) | | 56 | 22 | 70 | 41 | 89 | 67 | 78 | 63 | 59 | 93 | 81 |  |

Abbreviations: Y: yes; PY: partial yes; N: no.
