# Supplementary material for: Effects of exercise-based home pulmonary rehabilitation on patients with chronic obstructive pulmonary disease: An overview of systematic review
Source: PLoS One. 2022 Nov 17;17(11):e0277632. doi: 10.1371/journal.pone.0277632 (PMC9671331; doi:10.1371/journal.pone.0277632)
Supplement: S5 Table — (DOCX) [file pone.0277632.s005.docx]

**Supplementary Table 5.** **Results of evidence quality (based on the GRADE tool)**

| **First Author, year** | **Interventions vs. comparisons** | **Outcomes** | **Certainty assessment** | | | | | | | **Relative effect (95% CI)** | **P-value** | **Quality** |
| --- | --- | --- | --- | --- | --- | --- | --- | --- | --- | --- | --- | --- |
|  |  |  | **Design** | **Limitations** | **Inconsistency** | **Indirectness** | **Imprecision** | **Publication bias** | **Study numbers and sample size** |  |  |  |
| Wang (2013) [32] | Exercise-based HPR vs. usual care | 6MWD | RCT | serious^1^ | serious^2^ | NO | NO | NO | 6(229) | WMD 59.03 (50.23, 67.83) | ＜0.0001 | Low |
|  |  | CRQ-Dyspnea | RCT | serious^1^ | NR | NO | NO | NO | 6(213) | MD 0.78 (0.24 ~ 1.32) | ＜0.01 | Not evaluated |
|  |  | CRQ-Fatigue | RCT | serious^1^ | NR | NO | NO | NO | 6(213) | MD 1.12 (0.54 ~ 1.70) | ＜0.01 | Not evaluated |
|  |  | CRQ-Emotion | RCT | serious^1^ | NR | NO | NO | NO | 6(213) | MD 1.05 (0.51 ~ 1.60) | ＜0.01 | Not evaluated |
|  |  | CRQ-Mastery | RCT | serious^1^ | NR | NO | serious^3^ | NO | 5(174) | MD 0.68 (-0.10 ~ 1.46) | ＞0.05 | Not evaluated |
| Liu  (2014) [33] | Exercise-based HPR vs. conventional community care | SGRQ | RCT | serious^1^ | NO | NO | serious^3^ | NO | 3(112) | MD -11.33(-16.37, -6.29) | ＜0.0001 | Low |
|  |  | CRQ-Dyspnea | RCT | serious^1^ | NO | NO | serious^3^ | serious^4^ | 2(73) | MD -5.70(-8.60, -2.81) | NR | Very low |
|  |  | CRQ-Fatigue | RCT | serious^1^ | NO | NO | serious^3^ | serious^4^ | 2(73) | MD -3.15(-5.46, -0.83) | NR | Very low |
|  |  | CRQ-Emotion | RCT | serious^1^ | NO | NO | serious^3^ | serious^4^ | 2(73) | MD -4.76(-8.32, -1.21) | NR | Very low |
|  |  | CRQ-Mastery | RCT | serious^1^ | NO | NO | serious^3^ | serious^4^ | 2(73) | MD - 2.96(-5.29, -0.63) | NR | Very low |
|  |  | Borg | RCT | serious^1^ | NO | NO | serious^3^ | serious^4^ | 2(85) | MD -0.92(-1.61, -0.23) | 0.009 | Very low |
|  |  | 6MWD | RCT | serious^1^ | serious^2^ | NO | serious^3^ | NO | 4(167) | MD 35.88(9.38, 62.38) | 0.008 | Very low |
|  |  | FEV1/FVC | RCT | serious^1^ | serious^2^ | NO | serious^3^ | serious^4^ | 2(82) | MD -10.72(-15.86, -5.58) | < 0.0001 | Very low |
| Liu (2016) [34] | Exercise-based HPR vs. conventional community care | SGRQ | RCT | serious^1^ | NO | NO | serious^3^ | serious^4^ | 4(153) | MD -14.19(-18.27, -10.11) | ＜0.0001 | Very low |
|  |  | CRQ-Dyspnea | RCT | serious^1^ | serious^2^ | NO | serious^3^ | serious^4^ | 2(62) | MD 4.10(1.36, 6.85) | 0.003 | Very low |
|  |  | CRQ-Fatigue | RCT | serious^1^ | NO | NO | serious^3^ | serious^4^ | 2(62) | MD 5.14(2.74, 7.53) | ＜0.0001 | Very low |
|  |  | CRQ-Emotion | RCT | serious^1^ | NO | NO | serious^3^ | serious^4^ | 2(62) | MD 5.09(0.59, 9.59) | 0.03 | Very low |
|  |  | Borg | RCT | serious^1^ | NO | NO | serious^3^ | serious^4^ | 2(53) | MD -1.54(-2.73, -0.36) | 0.01 | Very low |
| Neves  (2016) [16] | Exercise-based HPR vs. no exercise | 6MWT | RCT | serious^1^ | serious^2^ | NO | serious^3^ | NO | 10(461) | MD 33.79(6.03, 61.54) | 0.02 | Very low |
|  |  | ISWT | RCT | serious^1^ | NO | NO | serious^3^ | NO | 7(321) | MD 73.84(43.04, 104.64) | ＜0.0001 | Low |
|  |  | MRC-dyspnea | RCT | serious^1^ | NO | NO | NO | NO | 4(280) | MD -0.20(-0.43, 0.00) | 0.06 | Moderate |
|  |  | CRQ-Fatigue | RCT | serious^1^ | NO | NO | NO | NO | 13(521) | MD 0.79(0.51, 1.07) | ＜0.0001 | Moderate |
|  |  | CRQ-Dyspnea | RCT | serious^1^ | serious^2^ | NO | NO | NO | 13(521) | MD 0.65(0.21, 1.09) | ＜0.004 | Low |
|  |  | CRQ-Emotion | RCT | serious^1^ | serious^2^ | NO | NO | NO | 13(521) | MD 0.51(0.20, 0.83) | 0.001 | Low |
|  |  | CRQ-Mastery | RCT | serious^1^ | serious^2^ | NO | NO | NO | 12(482) | MD 0.73(0.38, 1.08) | ＜0.0001 | Low |
|  |  | SGRQ | RCT | serious^1^ | serious^2^ | NO | serious^3^ | NO | 4(281) | MD -9.98(-17.23, -2.74) | 0.007 | Very low |
|  | Exercise-based HPR vs. OPR | 6MWT | RCT | serious^1^ | NO | NO | serious^3^ | NO | 3(334) | MD 8.70(-6.67, 24.08) | 0.27 | Low |
|  |  | CRQ-Dyspnea | RCT | serious^1^ | NO | NO | serious^3^ | serious^4^ | 3(123) | MD -0.14(-0.57, 0.29) | 0.54 | Very low |
|  |  | CRQ-Fatigue | RCT | serious^1^ | serious^2^ | NO | serious^3^ | serious^4^ | 3(123) | MD -0.18(-0.77, 0.42) | 0.56 | Very low |
|  |  | CRQ-Emotion | RCT | serious^1^ | serious^2^ | NO | serious^3^ | serious^4^ | 3(123) | MD -0.23(-0.99, 0.53) | 0.55 | Very low |
|  |  | CRQ-Mastery | RCT | serious^1^ | serious^2^ | NO | serious^3^ | serious^4^ | 3(123) | MD -0.14(-0.95, 0.68) | 0.74 | Very low |
| Li (2017) [35] | Exercise-based HPR vs. usual care | SGRQ | RCT | serious^1^ | NO | NO | serious^3^ | NO | 5(196) | MD -13.56(-17.56, -9.56) | ＜0.0001 | Low |
|  |  | 6MWD | RCT | serious^1^ | NO | NO | serious^3^ | NO | 6(241) | MD 48.23(25.60, 70.86) | ＜0.0001 | Low |
|  |  | SWT | RCT | serious^1^ | NO | NO | serious^3^ | serious^4^ | 2(87) | MD 39.29(-25.49, 104.07) | ＞0.05 | Very low |
|  |  | CRQ-Dyspnea | RCT | serious^1^ | NO | NO | serious^3^ | serious^4^ | 2(65) | MD -0.02 (-0.47, 0.43) | ＞0.05 | Very low |
|  |  | CRQ-Fatigue | RCT | serious^1^ | NO | NO | serious^3^ | serious^4^ | 2(65) | MD -0.08 (-0.61, 0.45) | ＞0.05 | Very low |
|  |  | CRQ-Emotion | RCT | serious^1^ | NO | NO | serious^3^ | serious^4^ | 2(65) | MD -0.28 (-0.82, 0.27) | ＞0.05 | Very low |
|  |  | CRQ-Mastery | RCT | serious^1^ | NO | NO | serious^3^ | serious^4^ | 2(65) | MD -0.23 (-0.86, 0.41) | ＞0.05 | Very low |
|  |  | Borg | RCT | serious^1^ | NO | NO | serious^3^ | serious^4^ | 3(117) | MD -1.03(-1.64, -0.42) | ＜0.05 | Very low |
|  |  | MRC | RCT | serious^1^ | NO | NO | serious^3^ | serious^4^ | 2(78) | MD -0.79(-1.16, -0.43) | ＜0.05 | Very low |
|  |  | FEV1/FVC | RCT | serious^1^ | NO | NO | serious^3^ | serious^4^ | 3(85) | MD -0.3(-6.43，5.83) | ＞0.05 | Very low |
| Wuytack  (2018) [36] | Exercise-based HPR vs. OPR | CRQ-Dyspnea | RCT | serious^1^ | NO | NO | NO | NO | 4(473) | MD -0.09(-0.28, 0.10) | 0.35 | Moderate |
|  |  | CRQ-Fatigue | RCT | serious^1^ | NO | NO | NO | NO | 4(473) | MD -0.00(-0.18, 0.17) | 0.99 | Moderate |
|  |  | CRQ-Emotion | RCT | serious^1^ | serious^2^ | NO | NO | NO | 4(473) | MD 0.10(-0.24, 0.45) | 0.56 | Low |
|  |  | CRQ-Mastery | RCT | serious^1^ | NO | NO | NO | NO | 4(473) | MD -0.02(-0.28, 0.25) | 0.89 | Moderate |
|  |  | SGRQ | RCT | serious^1^ | serious^2^ | NO | serious^3^ | serious^4^ | 2(283) | MD -0.82(-7.47, 5.83) | 0.81 | Very low |
|  |  | 6MWD | RCT | serious^1^ | serious^2^ | NO | serious^3^ | NO | 5(538) | MD -5.49(-24.51, 13.52) | 0.57 | Very low |
|  | Exercise-based community PR vs. OPR | CRQ-Dyspnea | RCT | serious^1^ | NO | NO | serious^3^ | serious^4^ | 2(195) | MD 0.29(-0.05, 0.62) | 0.10 | Very low |
|  |  | CRQ-Fatigue | RCT | serious^1^ | serious^2^ | NO | serious^3^ | serious^4^ | 2(200) | MD -0.02(-1.09, 1.05) | 0.97 | Very low |
|  |  | CRQ-Emotion | RCT | serious^1^ | NO | NO | serious^3^ | serious^4^ | 2(198) | MD 0.10(-0.40, 0.59) | 0.70 | Very low |
|  |  | CRQ-Mastery | RCT | serious^1^ | NO | NO | serious^3^ | serious^4^ | 2(198) | MD -0.08(-0.45, 0.28) | 0.65 | Very low |
| Chen  (2020) [37] | Exercise-based HPR vs. centre-based PR | 6MWT | RCT | serious^1^ | NO | NO | serious^3^ | NO | 7(811) | MD -1.64(-11.42, 8.15) | 0.74 | Low |
|  |  | EWST | RCT | serious^1^ | NO | NO | serious^3^ | serious^4^ | 2(198) | MD -51.95(-136.83, 32.94) | 0.23 | Very low |
|  |  | CRQ-Dyspnea | RCT | serious^1^ | NO | NO | NO | NO | 5(608) | MD -0.08(-0.30, 0.13) | 0.45 | Moderate |
|  |  | CRQ-Fatigue | RCT | serious^1^ | NO | NO | NO | NO | 4(549) | MD -0.19(-0.45, 0.07) | 0.14 | Moderate |
|  |  | CRQ-Emotion | RCT | serious^1^ | NO | NO | NO | NO | 4(537) | MD -0.18(-0.40, 0.04) | 0.11 | Moderate |
|  |  | CRQ-Mastery | RCT | serious^1^ | NO | NO | NO | NO | 4(547) | MD -0.13(-0.38, 0.11) | 0.30 | Moderate |
|  |  | SGRQ | RCT | serious^1^ | NO | NO | serious^3^ | serious^4^ | 2(274) | MD -1.77(-4.54, 0.99) | 0.21 | Very low |
|  |  | mMRC | RCT | serious^1^ | NO | NO | serious^3^ | serious^4^ | 2(238) | MD -0.15(-0.46, 0.17) | 0.35 | Very low |
| Fu (2021) [38] | Exercise-based HPR vs. standard medical care | FVC | RCT | serious^1^ | serious^2^ | NO | NO | NO | 8(748) | SMD 1.137(0.671, 1.603) | ＜0.05 | Low |
|  |  | FEV1 | RCT | serious^1^ | serious^2^ | NO | NO | NO | 14(1443) | SMD 1.338(0.731, 1.945) | ＜0.05 | Low |
|  |  | FEV1/FVC | RCT | serious^1^ | serious^2^ | NO | NO | NO | 15(1463) | SMD 1.91(1.26, 2.56) | ＜0.05 | Low |
|  |  | 6MWT | RCT | serious^1^ | serious^2^ | NO | NO | NO | 11(1163) | SMD 1.25(0.78, 1.71) | ＜0.05 | Low |
|  |  | SGRQ | RCT | serious^1^ | serious^2^ | NO | NO | NO | 8(723) | SMD -2.271(-3.171, -1.37) | ＜0.05 | Low |
| Mendes Xavier  (2022) [39] | Exercise-based HPR vs. conventional PR | CRQ-Dyspnea | RCT | serious^1^ | serious^2^ | NO | serious^3^ | NO | 4(223) | MD 0.31(-0.11, 0.74) | 0.33 | Very low |
|  |  | MRC/mMRC | RCT | serious^1^ | serious^2^ | NO | NO | NO | 5(457) | MD -0.55(-0.84, -0.26) | 0.30 | Low |
|  |  | 6MWT | RCT | serious^1^ | NO | NO | NO | NO | 13(764) | MD 51.20(37.32, 65.09) | 0.78 | Moderate |
|  |  | SGRQ | RCT | serious^1^ | NO | NO | NO | NO | 7(520) | MD -9.34(-13.25, -5.43) | 0.24 | Moderate |
| Paixão  (2022) [40] | Exercise-based HPR vs. usual care | CRQ-Dyspnea | RCT | serious^1^ | NO | NO | NO | NO | 4(480) | MD 0.12(0.09, 0.15) | ＜0.01 | Moderate |
|  |  | 6MWD | RCT | serious^1^ | serious^2^ | NO | serious^3^ | NO | 4(430) | MD 13.7(3.58, 23.83) | ＜0.01 | Very low |
|  |  | ISWD | RCT | serious^1^ | serious^2^ | NO | serious^3^ | NO | 3(216) | MD 58.59(5.79, 111.39) | 0.03 | Very low |

Abbreviations: 95% CI: 95% of confidence intervals; HPR: home pulmonary rehabilitation; 6MWD: 6-minute walk distance; RCT: randomized controlled trial; WMD: weighted mean difference; NR, not reported; MD: mean difference; SGRQ: St George’s respiratory questionnaire; CRQ: chronic respiratory disease questionnaire; Borg: Borg scale; 6MWT: 6-minute walk test; ISWT: incremental shuttle walk test; SWT: shuttle walk test; MRC: medical research council; OPR: outpatient pulmonary rehabilitation; FEV1: forced expiratory volume in 1 second; FVC: forced volume vital capacity; EWST: endurance shuttle walk test; mMRC: modified British Medical Research Council; ISWD: incremental shuttle walk distance;

Notes: 1: The experimental design had a large bias in random and distributive findings or was blind; 2: I^2^ value of the combined results was large, and confidence intervals overlapped difference; 3: The confidence interval was not narrow enough, or the sample size is too small; 4: Few studies are included, and there may be a large publication bias.
